# Vision-aided brain–machine interface training system for robotic arm control and clinical application on two patients with cervical spinal cord injury

**DOI:** 10.1186/s12938-019-0633-6

**Published:** 2019-02-11

**Authors:** Yoon Jae Kim, Hyung Seok Nam, Woo Hyung Lee, Han Gil Seo, Ja-Ho Leigh, Byung-Mo Oh, Moon Suk Bang, Sungwan Kim

**Affiliations:** 1 Interdisciplinary Program for Bioengineering, Graduate School, Seoul National University, Seoul, 08826 South Korea; 2 Department of Biomedical Engineering, Seoul National University College of Medicine, Seoul, 03080 South Korea; 3 Department of Rehabilitation Medicine, Seoul National University College of Medicine, Seoul, 03080 South Korea; 4 Department of Rehabilitation Medicine, Seoul National University Hospital, Seoul, 03080 South Korea; 5 Department of Rehabilitation Medicine, Incheon St. Mary’s Hospital, College of Medicine, The Catholic University of Korea, Incheon, 21431 South Korea; 6 Institute of Medical and Biological Engineering, Medical Research Center, Seoul National University, Seoul, 03080 South Korea

**Keywords:** Brain machine interface, Spinal cord injury, Electroencephalography, Functional magnetic resonance image

## Abstract

**Background:**

While spontaneous robotic arm control using motor imagery has been reported, most previous successful cases have used invasive approaches with advantages in spatial resolution. However, still many researchers continue to investigate methods for robotic arm control with noninvasive neural signal. Most of noninvasive control of robotic arm utilizes P300, steady state visually evoked potential, N2pc, and mental tasks differentiation. Even though these approaches demonstrated successful accuracy, they are limited in time efficiency and user intuition, and mostly require visual stimulation. Ultimately, velocity vector construction using electroencephalography activated by motion-related motor imagery can be considered as a substitution. In this study, a vision-aided brain–machine interface training system for robotic arm control is proposed and developed.

**Methods:**

The proposed system uses a Microsoft Kinect to detect and estimates the 3D positions of the possible target objects. The predicted velocity vector for robot arm input is compensated using the artificial potential to follow an intended one among the possible targets. Two participants with cervical spinal cord injury trained with the system to explore its possible effects.

**Results:**

In a situation with four possible targets, the proposed system significantly improved the distance error to the intended target compared to the unintended ones (*p *< 0.0001). Functional magnetic resonance imaging after five sessions of observation-based training with the developed system showed brain activation patterns with tendency of focusing to ipsilateral primary motor and sensory cortex, posterior parietal cortex, and contralateral cerebellum. However, shared control with blending parameter α less than 1 was not successful and success rate for touching an instructed target was less than the chance level (= 50%).

**Conclusions:**

The pilot clinical study utilizing the training system suggested potential beneficial effects in characterizing the brain activation patterns.

## Background

People can face losing all or part of their motor functions because of various diseases or physical accidents such as spinal cord injury, stroke, and amyotrophic lateral sclerosis. The damage to motor functions frequently makes it difficult to perform activities of daily living [1, 2]. Among various assistive technologies, brain–machine interfaces (BMIs), which depends on features from user’s neural signals, enables control of external devices. BMIs allow a person to bypass conventional neuromuscular pathways to interact with the environment.

Since the concept of BMIs was first proposed in the 1970s at the University of California Los Angeles [3, 4], scientists and engineers have improved upon technology for developing a human-controlled external robotic arm that does not require physical movement [5–8]. Monkeys have fed themselves by controlling a robotic arm [9], and humans have utilized invasive neural signals to control a seven degree-of-freedom (DOF) robotic arm as if it were their own [10, 11]. The previous notable studies [10, 11] showed that the success rate of reaching and grasping can reach approximately 70–90% in allocated time. These studies used intracortical microelectrode array (MEA), which are highly invasive. Features from spikes measured from MEA provide high spatial resolution, which is advantageous in accurate prediction of imagery hand velocity.

Even though approaches based on MEA are highly successful, many researchers still attempt to control a robotic arm with noninvasive neural signals, such as those derived from electroencephalography (EEG), which does not require surgically implanting an electrode array. Previous EEG studies for robotic arm control uses features, such as P300 [12–15], N2pc [13, 15], steady-state visual evoked potential [16–19], and mental task differentiation [13, 20–24]. These approaches have demonstrated high performance in terms of accuracy, but relatively unintuitive, inefficient in terms of time, and mostly requires additional interfaces for visual stimulation. Thus, hand velocity predicted from EEG activated by motion-related motor imagery can be considered as an ultimate substitution for conventional EEG based control. Even though some research using mental task differentiation [13, 20–24] exhibited robotic arm control based on motor imagery, they utilized classification approach rather than velocity vector prediction. Velocity control of robotic arm base on EEG activated by motor imagery is a highly challenging purpose and has not yet exhibited satisfactory performance because of its limited spatial resolution and low signal-to-noise ratio [25].

**Fig. 1:**
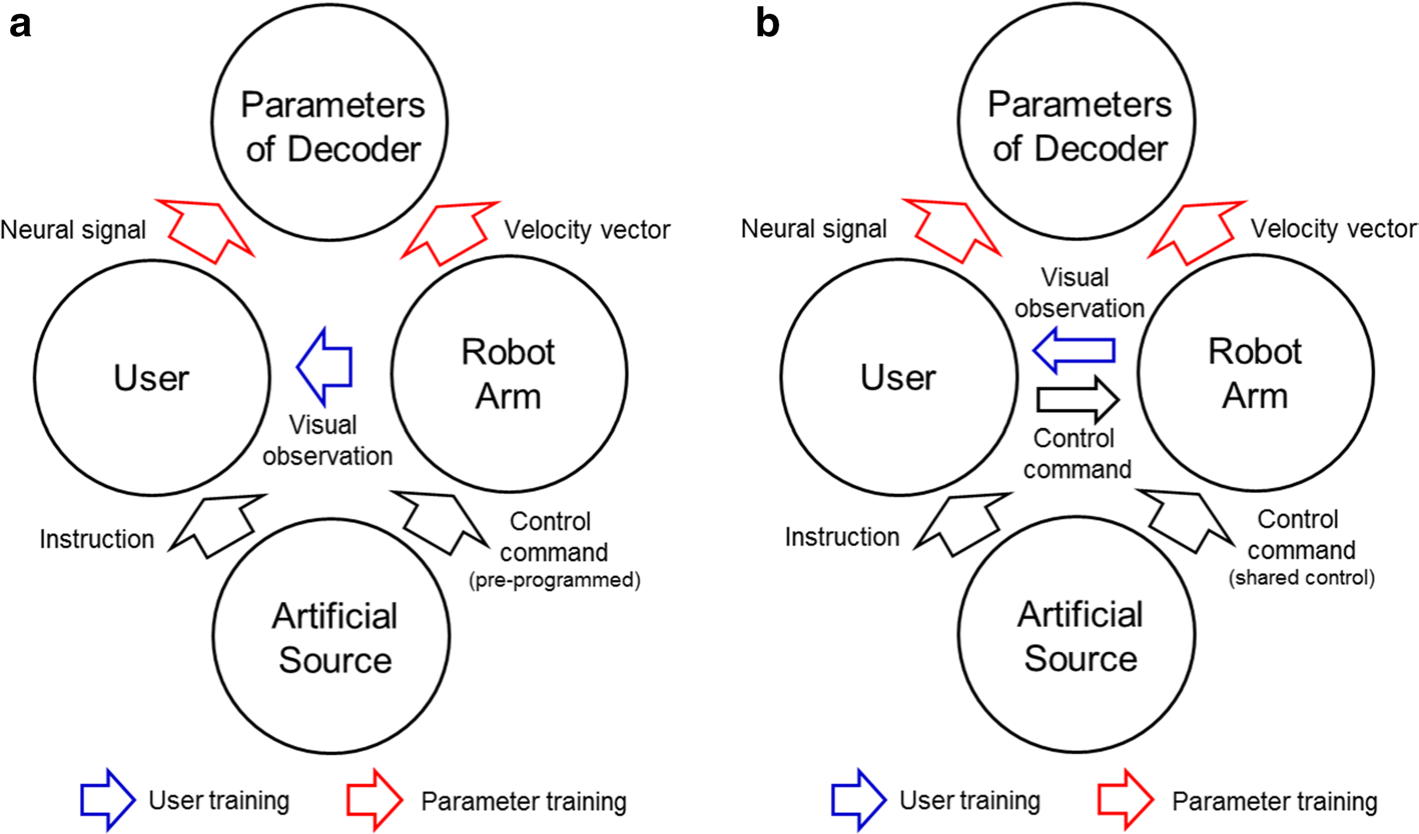
Two types of BMI training for robotic arm control. **a** Observation-based training. **b** Shared control based training. The training approaches consist of user training for the adaptation of motor imagery and parameter training for improved decoding performance

Thus, we propose and develop a novel training system, which can improve motor imagery ability to extract features for velocity vector prediction. The training system utilizes shared control which uses auxiliary camera and makes the control easier. Shared control approaches that blend sensor information and neural signals for a more intelligent system have been applied to BMI field for improvement of task performance or fine control of terminal device [26–29]. In the previous studies, the shared control approaches have been applied to determination of the parameters of decoder and user adaptation (co-adaptive process) [11, 30]. The studies were applied to train primates [30] and humans [11], and the training process can proceed in two phases. In the first phase, observation-based training is provided (Fig. 1a). During the phase, the robotic arm moves automatically driven by preprogrammed algorithm to reach targets and an initial decoder is generated. In the second phase of calibration, the user controls the robotic arm by using the initial decoder while the shared control strategy assists the reaching motion (Fig. 1b). An improved decoder can be built from the second phase. These studies utilized invasive MEA and the training process has not yet been applied to noninvasive neural signals to the best of our knowledge.

In this study, a novel vision-aided BMI training system is developed and applied to co-adaptive process of user and decoder. Whereas previous invasive studies used preferred direction based decoder, this study used regression between neural signal and hand velocity. The developed system is applied to two clinical cases of potential users (patients with cervical spinal cord injury) with functional magnetic resonance image (fMRI) studies to assess feasibility and possible effects of the training. As a neural signal, EEG is used because of its noninvasiveness.

## Methods

### Training system overview

The newly proposed training system automatically detects the target object on the basis of the image and uses the image information as well as the brain signal information to more easily control the robotic arm approaching the target object. During the phase of training with the system, shared control strategy can motivate user by maintaining the success rate to certain level. Kinect is used for image acquisition, and separation of pixel colour and hierarchical clustering is applied for multiple target object detection. For shared control, conventional artificial potential approach is modified to apply to BMI system for robotic arm control. As the dependency of image information is higher than that of brain signal information, the control difficulty of robotic arm is lowered. When the information from brain signal is completely suppressed, the robotic arm motion doesn’t reflect the user intention, and the process becomes an observation-based training. Further specific details are described in sections below.

### Detecting target objects using the Kinect

The position of target objects must be accurately defined to compensate for the predicted hand trajectory from neural signals. Before their position can be estimated, the targets must be detected. Although various target detection algorithms have been reported, elaborate algorithms are not required for the BMI training system because it is operated in a relatively well-arranged space with clean background. Green balls (diameter: 7 cm) serve as targets in this study, so binary images (green: 1; else: 0) were acquired using RGB images obtained by the Kinect (Kinect for XBOX 360, Microsoft, Redmond, WA, US). Using RGB values from the image, green pixels were separated, as shown in Eq. (1).

1 $$ \frac{\text{G}}{{{\text{R}} + {\text{G}} + {\text{B}}}} > 0.5 $$

The images were filtered to remove noise via the process described in Eqs. (2) and (3).

2 $$ {\text{Average }}\left( {{\text{i}}, {\text{j}}} \right) = \frac{1}{9}\mathop \sum \limits_{k = - 1}^{1} \mathop \sum \limits_{l = - 1}^{1} index\quad \left( {i + k,j + l} \right) $$

3 $$ \begin{aligned} {\text{Filtered}}\;\;{\text{pixel }}\left( {{\text{i}}, {\text{j}}} \right) & = 1 \quad \left( {{\text{if}}\;\;{\text{Average }}\left( {{\text{i}},{\text{j}}} \right) = 1} \right) \hfill \\ & = 0  \quad \left( {\text{else}} \right) \hfill \\ \end{aligned} $$

**Fig. 2:**
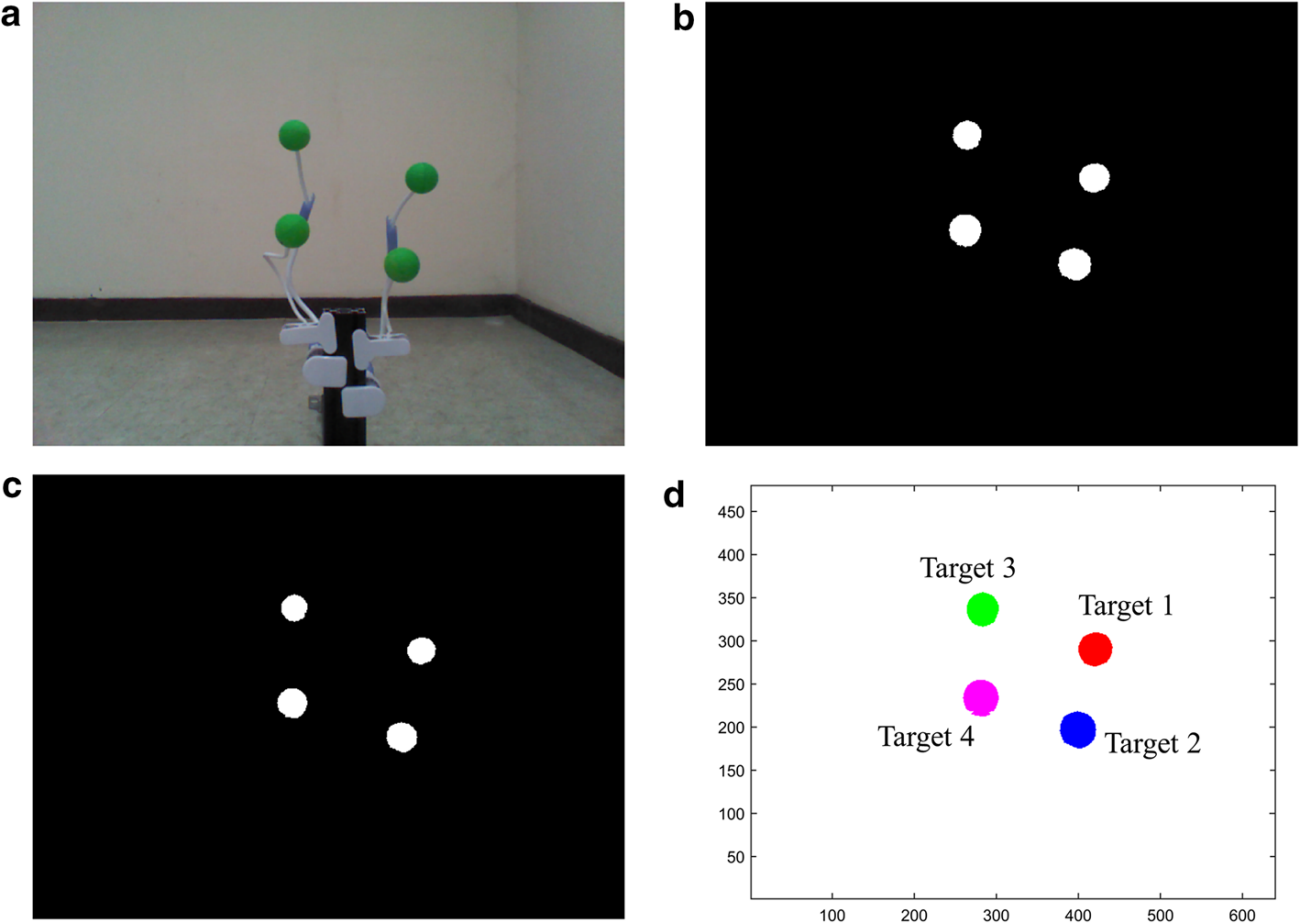
Procedure for target object detection. **a** RGB image obtained from Kinect. **b** Binary image. **c** Noise-filtered image. **d** Object detection via hierarchical clustering

Noise-filtered images can contain more than one target object, so pixels designated as “1” should be clustered to their corresponding target object. Hierarchical clustering distinguishes multiple target objects simultaneously. Conventional clustering algorithms require the number of clusters to be predetermined for centroid generation. However, a BMI system is highly limited if the number of target object is predetermined; thus, a divisive hierarchical clustering approach was chosen instead. This approach initially assumes that there is one target object is. When the x- or y-axis standard deviation of pixels are equal or larger than 20, the cluster is reclustered with two centroids. This procedure is repeated until all clusters have a pixel distribution whose standard deviation is less than 20 in both the x- and y-axes. The target detection procedure is summarized in Fig. 2.

To validate the algorithm, an environment containing several green target objects was prepared and photographed with the Kinect. The number of target objects ranged from two to four and 20 images were acquired for each number of target objects (for a total of 60 images). The distance between target objects was controlled within the range from 15 to 35 cm. For each number of target objects, the accuracy of detecting all existing targets was measured.

### Estimation of 3D position (camera calibration)

The 3D position of detected target object should be estimated using information acquired from images. The Kinect provides two types of images: RGB and depth images. RGB images provide three channels of data with 480 × 640 resolution. Depth images provide one channel of 480 × 640 resolution; each pixel represents a depth index related to the distance to objects in the image. Using three types of camera calibration, the 3D position of each target object can be estimated.

**Fig. 3:**
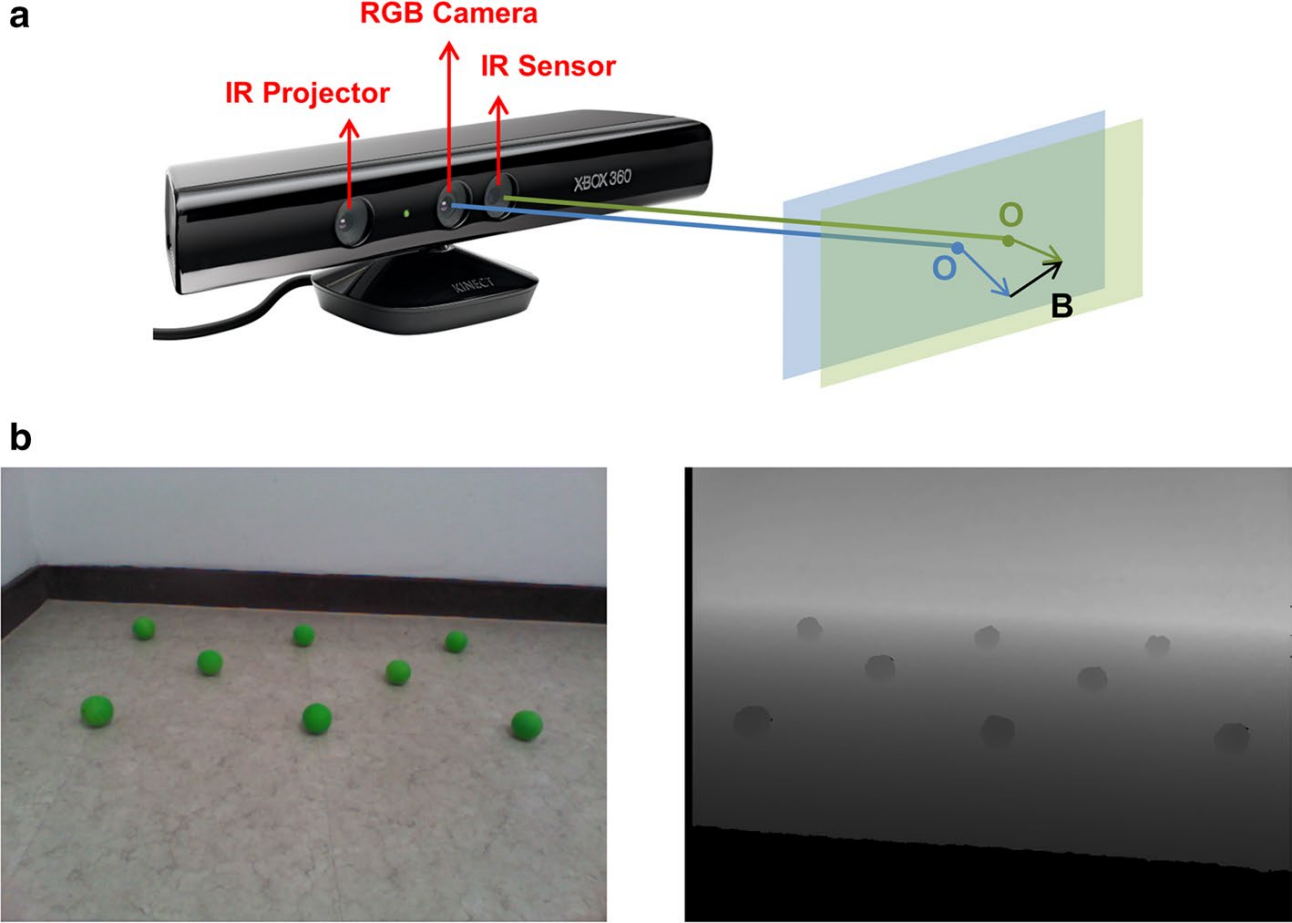
**a** Distortion between RGB and depth images caused by location of the sensors. **b** The same image photographed by the RGB (left) and depth (right) cameras, respectively

The first calibration consists of distortion compensation between the RGB and depth images. The same object is reflected in different pixels in the RGB and depth images, so calibration to match the two images is necessary for position estimation (Fig. 3a). Linear regression was applied to obtain the transformation matrix for the mapping. Images with several balls were photographed with the RGB and depth camera, respectively (Fig. 3b), and the pixel coordinates of the balls were measured. This process was repeated to obtain a larger dataset. The coordinates of the balls from the RGB images were stored to matrix A with a size of 165 × 2, which contains pixel information from 165 balls. Matrix D, for the depth image, was obtained similarly. Then, matrix B, which maps pixels from the RGB to the depth image, can be obtained with the linear regression shown below.

4 $$ {\text{D}} = \left[ {{\text{A }}1} \right] \times {\text{B}} $$

5 $$ {\text{B}} = \left( {X^{T} {\text{X}}} \right)^{ - 1} X^{T} D,         {\text{X}} = \left[ {{\text{A }}1} \right] $$

Thus, B was obtained as shown below. The R^2^ values for the x- and y-axes were nearly 1.0 (> 0.999) and their root mean square errors (RMSEs) were 2.97 and 2.40 pixels, respectively. This result indicates that less than 3 pixels of error are occurred with the proposed linear regression. When total size of the images (480 × 640 pixels) is considered, this error seems to be trivial.

6 $$ {\text{B}} = \left[ {\begin{array}{*{20}c} {1.17332} & {0.000815} \\ { - 0.01963} & {1.12255} \\ { - 34.6797} & { - 20.0941} \\ \end{array} } \right] $$

The second calibration is between the depth index and real distance. We utilized a linear curve fitting provided by the Matlab Curve Fitting Toolbox (Matlab R2016b, Mathworks Inc., Natick, MA, US), the result of which is shown in Eq. (7).

7 $$ Distance \, \left( {\text{m}} \right) = 61.5 \times Depth\;\;index + 0.1046,\;\; \left( {R^{2} = 0.9987} \right) $$

Finally, 2D position was estimated from the x and y pixels of the RGB image. The above approach was used and the results are shown in Eqs. (8) and (9).

8 $$ {\text{X}} \, \left( {\text{m}} \right) = \left( {0.001937 \times Distance \, \left( {\text{m}} \right) + 0.0001662} \right) \times Pixel,\;\; \left( {R^{2} = 0.9939} \right) $$

9 $$ {\text{Y}} \, \left( {\text{m}} \right) = \left( {0.002072 \times Distance \, \left( {\text{m}} \right) - 0.000227} \right) \times Pixel,\;\; \left( {R^{2} = 0.9784} \right) $$

**Fig. 4:**
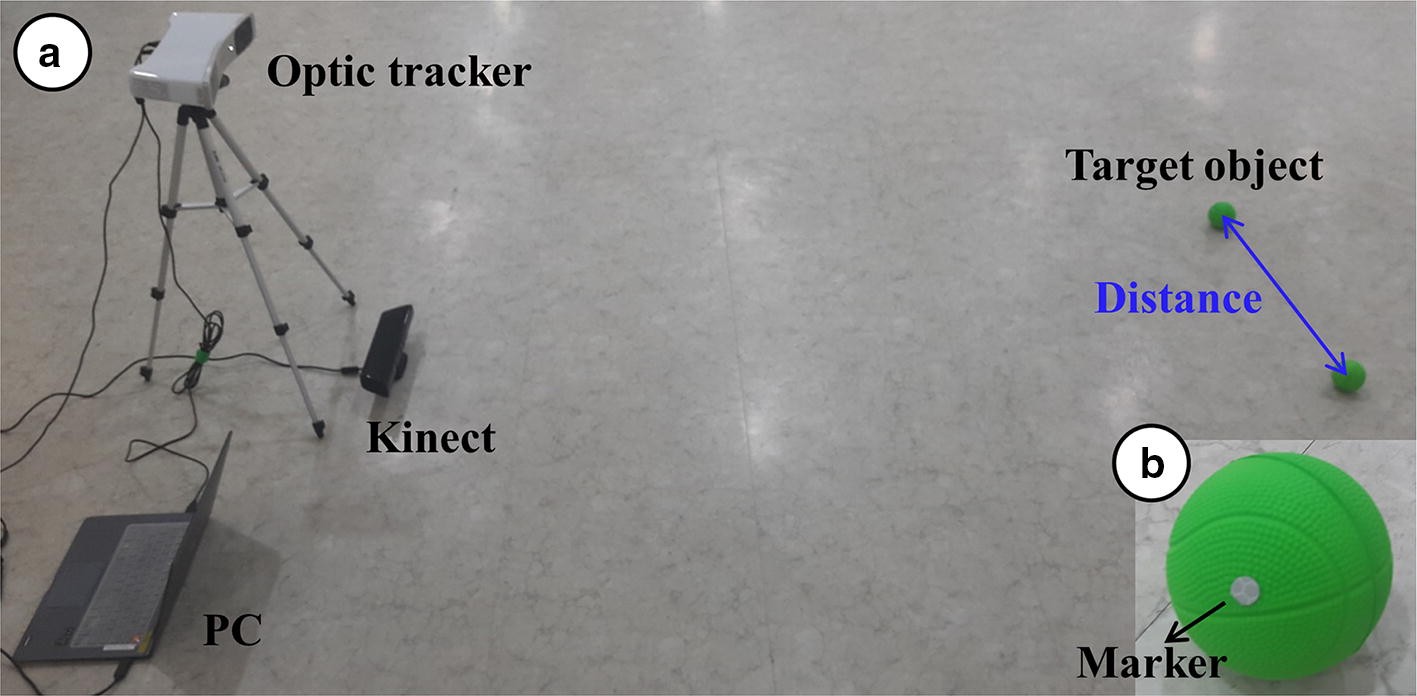
Experimental setup to validate camera calibration for position estimation

To validate the implemented algorithm, we compared it with an optic tracker (PST Base, ps-tech, Amsterdam, Netherlands) that estimates the 3D position of pre-attached stickers with high accuracy (positon < 0.5 mm RMSE, orientation < 1° RMSE^2^). Two target objects with pre-attached stickers were prepared and placed randomly on the prepared experimental setup, as depicted in Fig. 4. The distance between the two target objects was measured using the optic tracker and Kinect. This procedure was repeated 10 times and the difference between the two approaches was analyzed.

### Vision-aided robotic system hardware

**Fig. 5:**
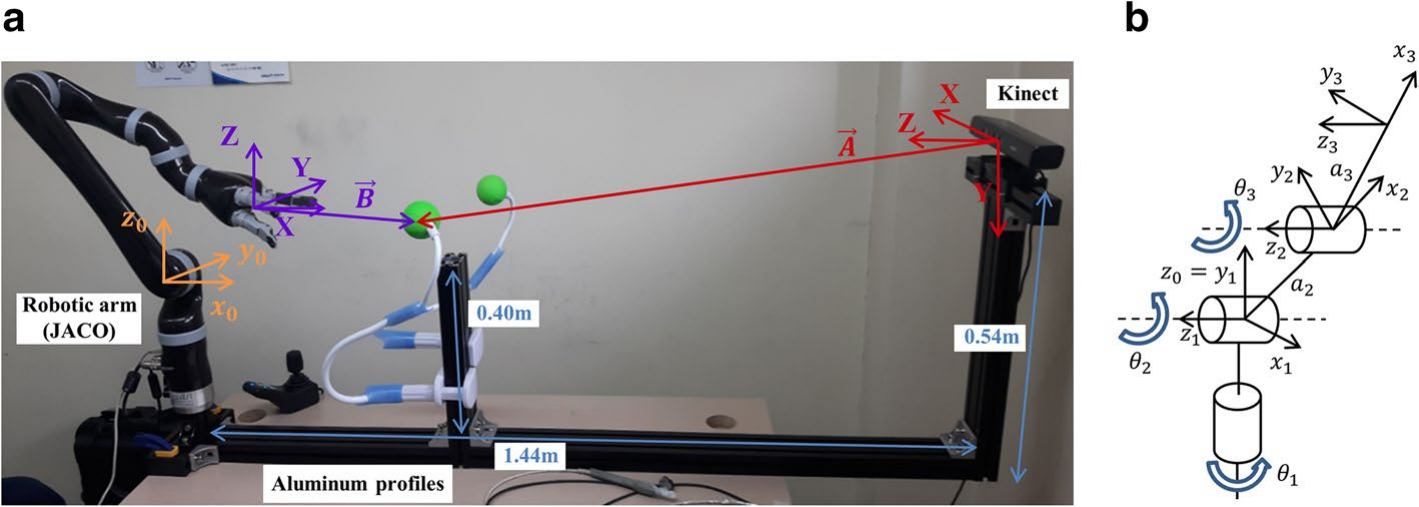
**a** BMI training system, consisting of a robotic arm, Kinect, and aluminum profiles. **b** Frames and variables of the robotic arm kinematics

The vision-aided robotic system consists of three components: a 6-DOF anthropomorphic robot arm (JACO, Kinova, Boisbriand, QC, Canada), Kinect, and targets. The dimensions of the aluminum profiles for fixing three components were designed by considering the workspace of the robotic arm, as shown in Fig. 5a. The arm was fixed on one side of the aluminum profiles and the Kinect on the other. The green target objects were fixed between them using flexible supports. Kinect detects target objects and the estimated positions are delivered to the robot arm for its waypoint generation. The coordinates of the Kinect and robot arm are different; thus, the homogeneous transformation matrix (Eqs. (10) and (11)) should be multiplied before the target position information is used for waypoint generation.

10 $$ \left[ {\begin{array}{*{20}c} {\vec{B}} \\ 1 \\ \end{array} } \right] = T\left[ {\begin{array}{*{20}c} {\vec{A}} \\ 1 \\ \end{array} } \right] $$

11 $$ {\text{T}} = \left[ {\begin{array}{*{20}c} R & {\begin{array}{*{20}c} {1.24} \\ 0 \\ {0.05} \\ \end{array} } \\ {\begin{array}{*{20}c} 0 & 0 & 0 \\ \end{array} } & 1 \\ \end{array} } \right],   \;\; \left( {R = \left[ {\begin{array}{*{20}c} 0 & 0 & { - 1} \\ 1 & 0 & 0 \\ 0 & { - 1} & 0 \\ \end{array} } \right]} \right) $$

Vectors $\vec{A}$ and $\vec{B}$ are the position of target object measured from the Kinect and robot gripper, respectively. The origin of the robotic arm was defined as a point with an offset of 0.2 m in the x- and z-axes from the origin of frame 0 (the center of joint1, see Fig. 5b).

### Shared control using artificial potential

To generate waypoints of robot operation, both neural signals and the target object location should be considered. Previous studies reported shared control blending a velocity vector predicted from neural signals and the position of a target object; however, none considers the highly practical situation in which more than one target object exists in the workspace to the best of our knowledge. Thus, it is necessary to propose a novel shared-control strategy for BMI system that is practical for use with multiple targets. To solve this issue, our research utilized artificial potential, which is a conventional motion planning approach for robots to avoid obstacles and reach a destination. In this study, we modified the conventional approach to a proper form to apply to the BMI training system. It attracts robots to the most probable target object that allows the robot end-effector to approach the user-intended target. In this study, the joints of the robotic arm are compensated by blending the predicted hand velocity and the ideal vector to the intended target.

The detailed algorithm for shared control is described below. First, artificial potential was formed by considering the intended target object using Eqs. (12)–(14).

12 $$ e_{i} = q_{g, i} - q         \quad \left( {{\text{i}} = 1, 2, 3, \ldots , {\text{Number of target objects}}} \right) $$

13 $$ U_{a, i} = \frac{1}{2}k_{a, i} e_{i}^{T} \left( q \right)e_{i} \left( q \right) $$

14 $$ f_{t} = \mathop \sum \limits_{i = 1}^{4} f_{a, i} \quad    \left( {f_{a,i} = k_{a, i} e_{i} \left( {\text{q}} \right)} \right) $$

$q$ is the configuration of the joints and $q_{g, i}$ is the goal configuration for the $i{\text{th}}$ target object. $U_{a, i}$ is the artificial potential provided by the $i{\text{th}}$ target object. The stiffness $k_{a, i}$ is programmed to be 1 when the user of the BMI system most intends to reach the $i{\text{th}}$ target object. The most intended target object is determined by the currently predicted velocity from neural signals. The angle between the predicted velocity vector and the vector from the current position to each target position is calculated and the target with the smallest angle is determined to be the intended target object. For the unselected target objects, $k_{a, i}$ is set to 0. So, $f_{t}$ is the attractive force acted by the intended target. The intended target object is updated for each stage of waypoint generation. Thus, the BMI user can change the preferred target object while controlling the robotic arm. Then, the ideal vector to the intended target object is generated based on the current waypoint of end-effector and attractive force $f_{t}$ as shown in Eq. (15).

15 $$ \Delta x_{i, k} = x_{e} \left( {q_{k} + f_{t} \left( {q_{k} } \right)} \right) - x_{e} \left( {q_{k} } \right) $$

$x_{e} \left( {q_{k} } \right)$ is the $k{\text {th}}$ waypoint for robot arm end-effector and the vector $\Delta x_{i, k}$ points the ideal direction to approach the intended target. When the predicted hand velocity vector moves away from the origin and the angle to the most intended target object is less than 90°, the next waypoint is generated via Eqs. (16) and (17).

16 $$ \Delta x_{k} = 1.5\left\{ {\alpha \left( {\Delta x_{i, k} \frac{{\Delta x_{n, k} }}{{\Delta x_{i, k} }}} \right) + \left( {1 - \alpha } \right)\Delta x_{n, k} } \right\} $$

17 $$ x_{e} \left( {q_{k + 1} } \right) = x_{e} \left( {q_{k} } \right) + \beta \Delta x_{k + 1} + \left( {1 - \beta } \right)\Delta x_{k} $$

$\Delta x_{k}$ is the compensation vector for the $k{\text{th}}$ waypoint and $\Delta x_{n, k}$ is the hand velocity vector predicted from neural signals. The vector $\Delta x_{i, k}$, which points in the ideal direction to the intended target, is scaled to the size of vector $\Delta x_{n, k}$, and the two are blended with proportion of $\alpha$ and $1 - \alpha$. The larger $\alpha$ increases the compensation from the Kinect and decreases the strength of the originally predicted hand velocity from neural signals. Additionally, inertia is considered via parameter $\beta$ to suppress unintended sudden movement of the robotic arm. Similar BMI studies of shared control also considered the issue of reducing acceleration, applying a smoothing approach to motion planning [31]. At Eq. (17), the compensation for the $k + 1{\text{th}}$ waypoint is provided by blending the $k{\text{th}}$ compensation vector with the proportion of $1 - \beta$. Blending parameters $\alpha$ and $\beta$ both range from 0 to 1.

To calculate equations for artificial potential, forward and inverse kinematics of the robotic arm are required; conventional kinematics of a 3-DOF anthropomorphic arm [32] were utilized in this study. The homogeneous transformation matrix for forward kinematics is suggested in Eq. (18). The Denavit–Hartenberg parameters for deriving the matrix are listed in Table 1.

**Table 1:** Denavit–Hartenberg parameters for the robotic arm

Link	*a*_*i*_	*a*_*i*_	*d*_*i*_	$\theta_{i}$
1	0	π/2	0	$\theta_{1}$
2	$a_{2} = 0.41\;{\text{m}}$	0	0	$\theta_{2}$
3	$a_{3} = 0.44\;{\text{m}}$	0	0	$\theta_{3}$

18 $$ T_{forward} \left( q \right) = \left[ {\begin{array}{*{20}c} {\begin{array}{*{20}c} {c_{1} c_{23} } & { - c_{1} s_{23} } \\ {s_{1} c_{23} } & { - s_{1} s_{23} } \\ \end{array} } & {\begin{array}{*{20}c} {s_{1} } & {c_{1} \left( {a_{2} c_{2} + a_{3} c_{23} } \right)} \\ { - c_{1} } & {s_{1} \left( {a_{2} c_{2} + a_{3} c_{23} } \right)} \\ \end{array} } \\ {\begin{array}{*{20}c} {s_{23} } & {       c_{23} } \\ 0 & {       0} \\ \end{array} } & {\begin{array}{*{20}c} 0 & {       a_{2} s_{2} + a_{3} s_{23} } \\ 0 & 1 \\ \end{array} } \\ \end{array} } \right] $$

Relevant nomenclature is suggested in Fig. 4b. $c_{1}$, $c_{2}$, and $c_{3}$ indicate ${ \cos }\theta_{1}$, ${ \cos }\theta_{2}$, and ${ \cos }\theta_{3}$. $s_{1}$, $s_{2}$, and $s_{3}$ indicate ${ \sin }\theta_{1}$, ${ \cos }\theta_{2}$, and ${ \sin }\theta_{3}$. Furthermore, $c_{23}$ and $s_{23}$ indicate ${ \cos }\left( {\theta_{2} + \theta_{3} } \right)$ and ${ \sin }\left( {\theta_{2} + \theta_{3} } \right)$, respectively. Additional information for inverse kinematics is suggested in Eqs. (19)–(21).

19 $$ \theta_{3} = {\text{atan}}2\left( {s_{3} , c_{3} } \right) $$

20 $$ \theta_{2} = {\text{atan}}2\left( {\left( {a_{2} + a_{3} c_{3} } \right)pW_{z} - a_{3} s_{3} \sqrt {pW_{x}^{2} + pW_{y}^{2} } , \;\;\;\;\left( {a_{2} + a_{3} c_{3} } \right)\sqrt {pW_{x}^{2} + pW_{y}^{2} } + a_{3} s_{3} pW_{z} } \right) $$

21 $$ \theta_{1} = {\text{atan}}2\left( {pW_{y} , pW_{x} } \right) $$

$pW_{x}$, $pW_{y}$, and $pW_{z}$ indicate the x-, y-, and z-coordinates of the end-effector position measured in frame 0.

The proposed algorithm was validated by applying it to hand trajectories predicted from noninvasive EEG signals. Predicted hand trajectories obtained in previous research [25] were used. The details of decoding process were introduced in the previous paper. The dataset contains 120 hand trajectories predicted from a healthy participant consisting of 4 directional reaching movements (30 trials per direction). Blending parameters $\alpha$ and $\beta$ affect the performance of the algorithm and were modulated from 0.05 to 1.00 in intervals of 0.05 for optimization.

### Clinical application in participants with upper limb impairment

**Fig. 6:**
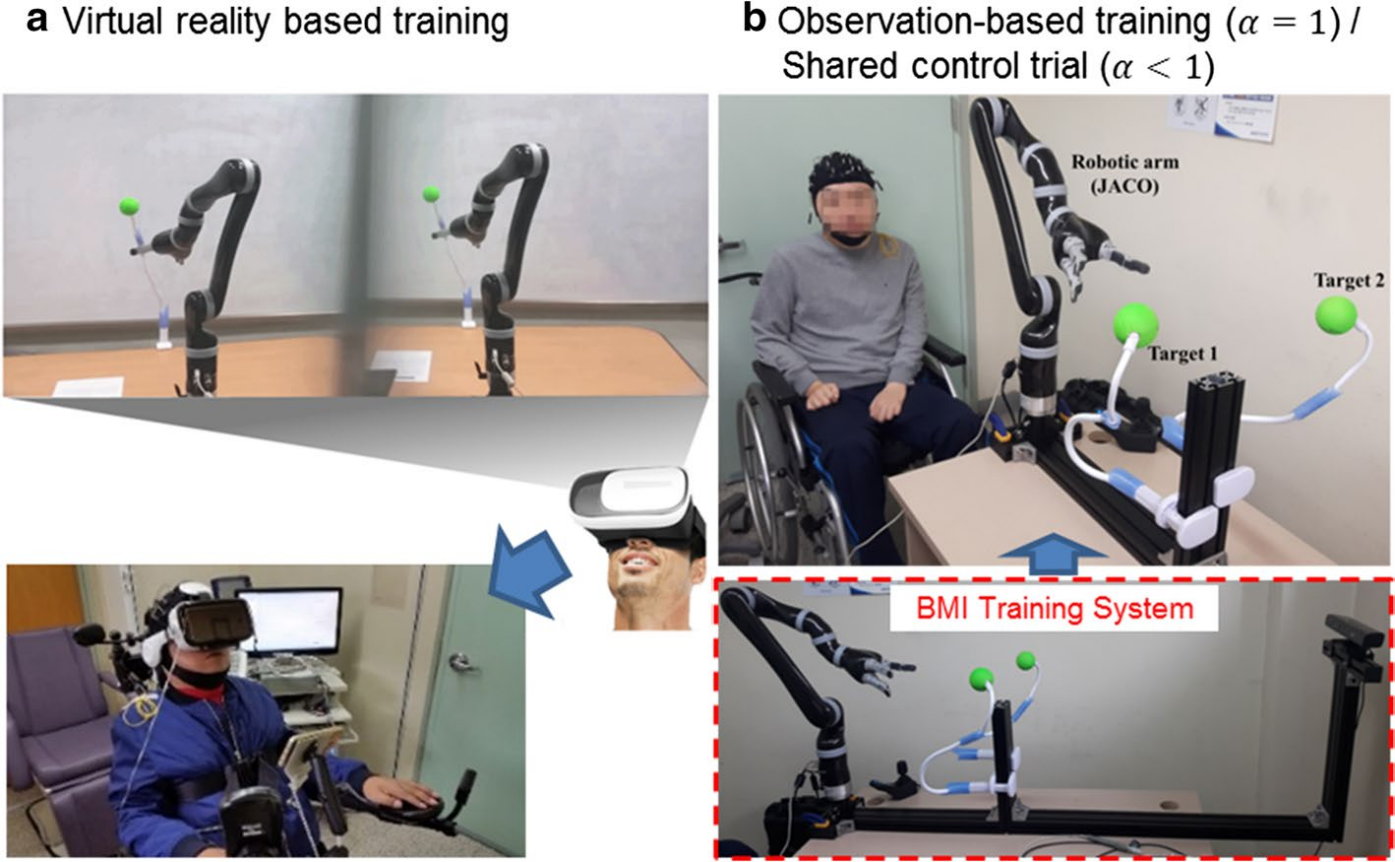
Training setup. **a** Virtual reality based training for the first 5 sessions. **b** Observation-based training for the last 5 sessions. After each observation-based training, shared control based reaching target was attempted to confirm improved decoding performance

**Fig. 7:**
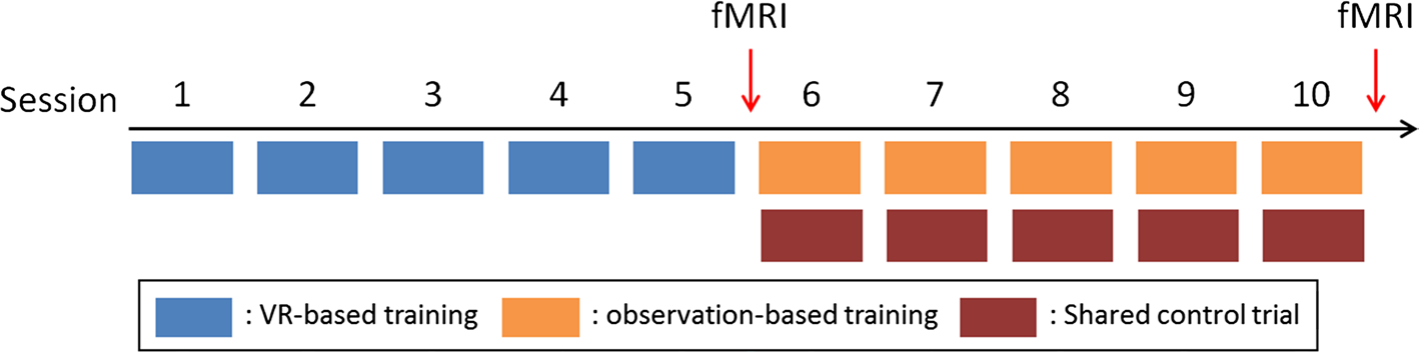
Overall plan of clinical application. Each participant participated the training session two times per a week, and it took about 5 weeks to complete the training processes

We established a clinical BMI training system consisting of an EEG acquisition system (Synamps 2, Compumedics Neuroscan, Texas, USA) and the vision-aided robotic system. The two systems are connected to a personal computer for a cooperated system. EEG is processed by Matlab, and the robotic arm is controlled by C# (Microsoft, Redmond, WA, USA) based SDK. Two volunteers with severe upper limb impairment due to cervical spinal cord injury participated in this study. The inclusion criteria for recruiting participants were: (1) severely disabled patient with unilateral or bilateral upper limb paralysis, (2) no significant cognitive impairment (MMSE > 26), and (3) able to fully understand the study and provide informed consent. Participant #1 is a 31-year-old male with American Spinal Injury Association Impairment Scale (AIS) C at level C4 (motor C7/C4 and sensory C5/C5), and participant #2 is a 47-year-old male with AIS B at level C4 (motor C4/C4 and sensory C5/C5). These patients are completely unable to control their own arms. The participants were given a total of 10 sessions for BMI training. The first 5 sessions were designed to help the participants get used to motor imagery using targets and virtual reality video files (Fig. 6a). In the next 5 sessions, developed BMI training system was utilized (Fig. 6b). In each session, the users were instructed to imagine robotic arm control to reach an instructed target out of two. Two types of training were applied to each session. As the first type, observation-based training was performed, and the parameters of the decoder were determined using multiple linear regressions between robotic arm motion and EEG signal. Since the decoder was not yet verified by patients with upper limb paralysis, blending parameters were set to 1, and the instructed targets were preprogrammed in the controller. After observation-based training of each session, shared control based robotic arm control was attempted with blending parameters α = β = 0.6. For each trial of shared control session, users were instructed to choose one target out of two, and success rate was measured for about 40 trials. fMRI while performing motor imagery tasks was taken before the 6th training session and after the 10th training session. Overall plan of clinical application is represented in Fig. 7. This clinical study was approved by the Institutional Review Board of Seoul National University Hospital (IRB No. 1605-136-765).

### fMRI evaluation

Functional imaging consisted of motor imagery tasks in 3 directions: upper, lower-left, and lower-right. Block design was used in all tasks; during each task, participants were instructed to imagine reaching and grasping movements repeatedly in selected directions. For each task, 8 active blocks and 7 rest blocks (each 20 s) were interleaved. The fMRI images were acquired with a Siemens MAGNETOM Trio, A Tim Syngo scanner using echo planar imaging (EPI, TE = 30 ms, TR = 3000 ms), angulated in parallel to the anterior and posterior commissure line. A T1-weighted image was also obtained for anatomical reference. The fMRI data were preprocessed using Statistical Parametric Mapping 12 (SPM12, Wellcome Trust Centre for Neuroimaging, London, UK; http://www.fil.ion.ucl.ac.uk/spm/) executed in MATLAB 2015b (Mathworks Inc., Natick, MA).

## Results

### Accuracy of target object detection and position estimation

No failure in accurate target detection occurred. In all images, the appropriate number of targets were detected at each location. The same conditions with two target objects were photographed and the distance between the two targets was measured using both the self-calibrated Kinect and an optic tracker. The optic tracker was used as a gold standard because it had been validated as a highly accurate system. In the second approach, as represented in Table 2, the Kinect-based system exhibited a distance error of 0.0250 ± 0.0160 m compared with the optic tracker. This distance error is 4.620 ± 3.490% of the total distance between the two target objects, indicating that there is less than a 2-cm error when the robot conducts approximately 40 cm of reaching and grasping movements. When the size of the robot gripper and the diameter of the target are considered, deviation of 1–2 cm is tolerable.

**Table 2:** Accuracy of the position estimation

No.	Distance measured using optic tracker (m)	Distance error (m)	Error percentage (%)
1	0.489	0.0158	3.221
2	0.589	0.0003	0.045
3	0.669	0.0197	2.949
4	0.430	0.0397	9.239
5	0.596	0.0401	6.725
6	0.572	0.0490	8.578
7	1.018	0.0238	2.335
8	1.013	0.0069	0.686
9	0.410	0.0373	9.106
10	0.519	0.0172	3.320
Average	0.630 ± 0.217	0.0250 ± 0.0160	4.620 ± 3.490

### Accuracy improvement of BMI system using artificial potential

**Fig. 8:**
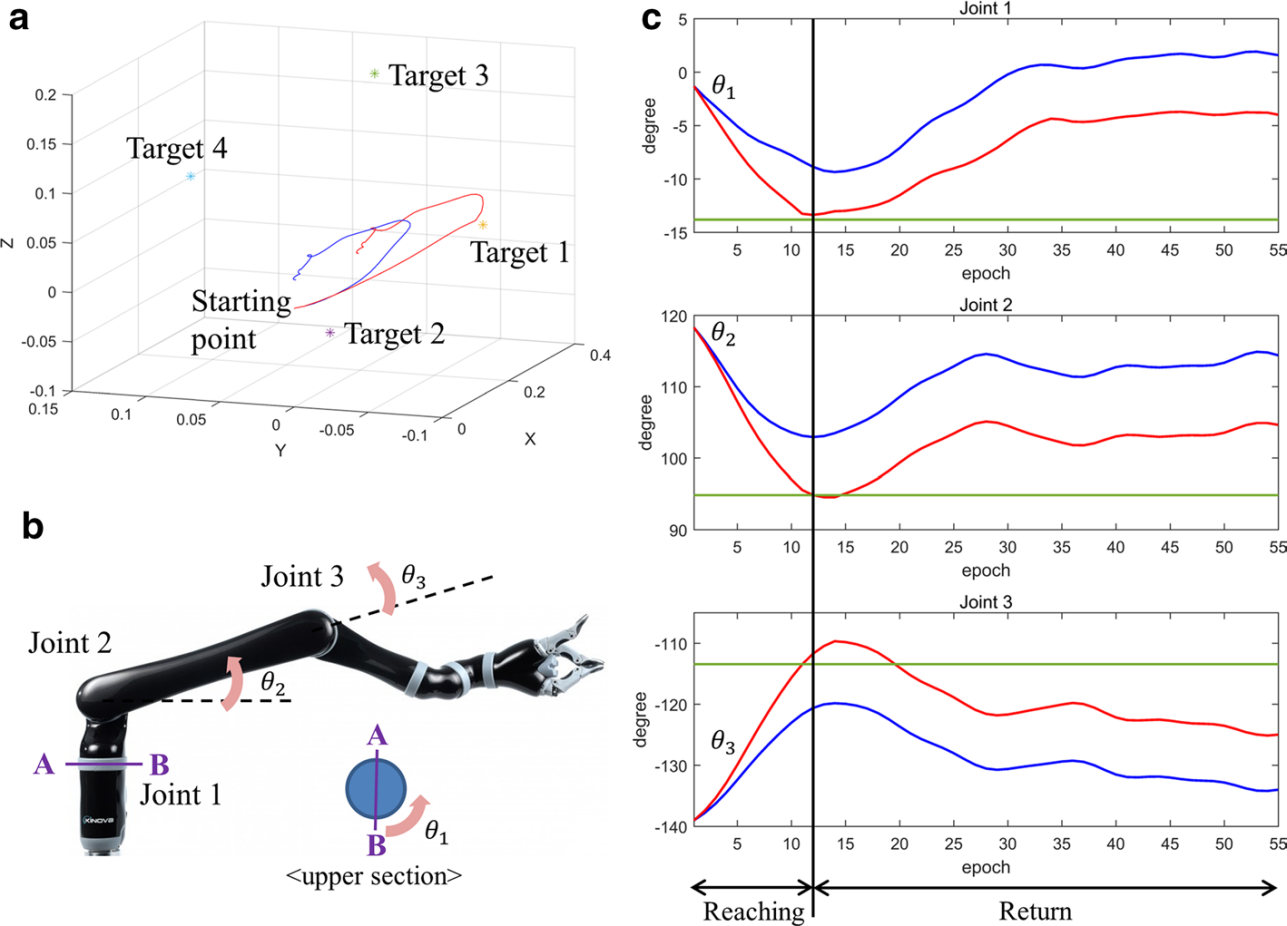
**a** Compensated trajectory (red) reaches more closely to the target object than the raw hand trajectory predicted from EEG. Blue line indicates trajectory with no compensation. **b** Definition of the joint angles of the robotic arm. **c** Green line indicates the ideal joint angle to reach the target object. Joint angles are closer to the ideal joint angles with compensation (red) than without (blue)

**Fig. 9:**
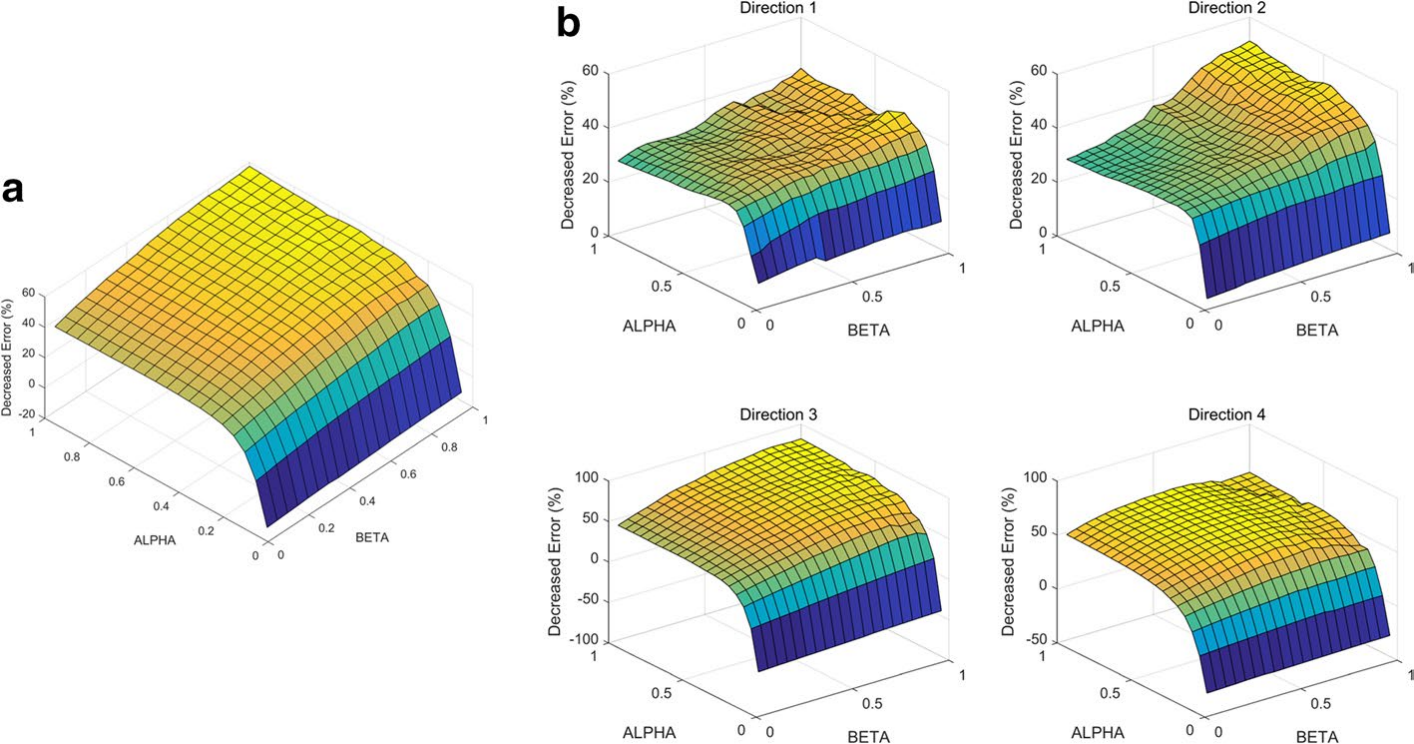
**a** Decreased error to the intended target averaged over all 120 trials. **b** Decreased error to intended target averaged over 30 trials for individual directions. The surface graph has a larger value from blue to yellow

This study proposes a shared control algorithm using artificial potential and validates it using hand trajectories predicted from noninvasive EEG neural signals. The algorithm was applied to 120 reaching trajectories in 4 directions. The target object positions were calculated using real hand trajectories, which were simultaneously measured with an accelerometer placed on the index finger. Applying this algorithm improved the predicted hand trajectories, and the shortest distance to the intended target object decreased, as shown in Fig. 8a. Additionally, we can confirm that the three joint angles of the robotic arm continuously reached to the ideally required joint angle to reach the target (Fig. 8b, c). Figure 9a shows the average improvement of the shortest distance to the target object as blending parameters differ. As blending parameters α and β increased, the degree of improvement increased the shortest distance to the target improved 57.37% when both blending parameters were 1.00. The patterns of improvement were similar for four each direction (Fig. 9b). Additionally, it was also confirm that the algorithm with low blending parameter α worsen the performance of the BMI system. The decreased error lower than 0 in Fig. 9 indicates the shortest distance to intended target object became farther rather than the improvement.

**Fig. 10:**
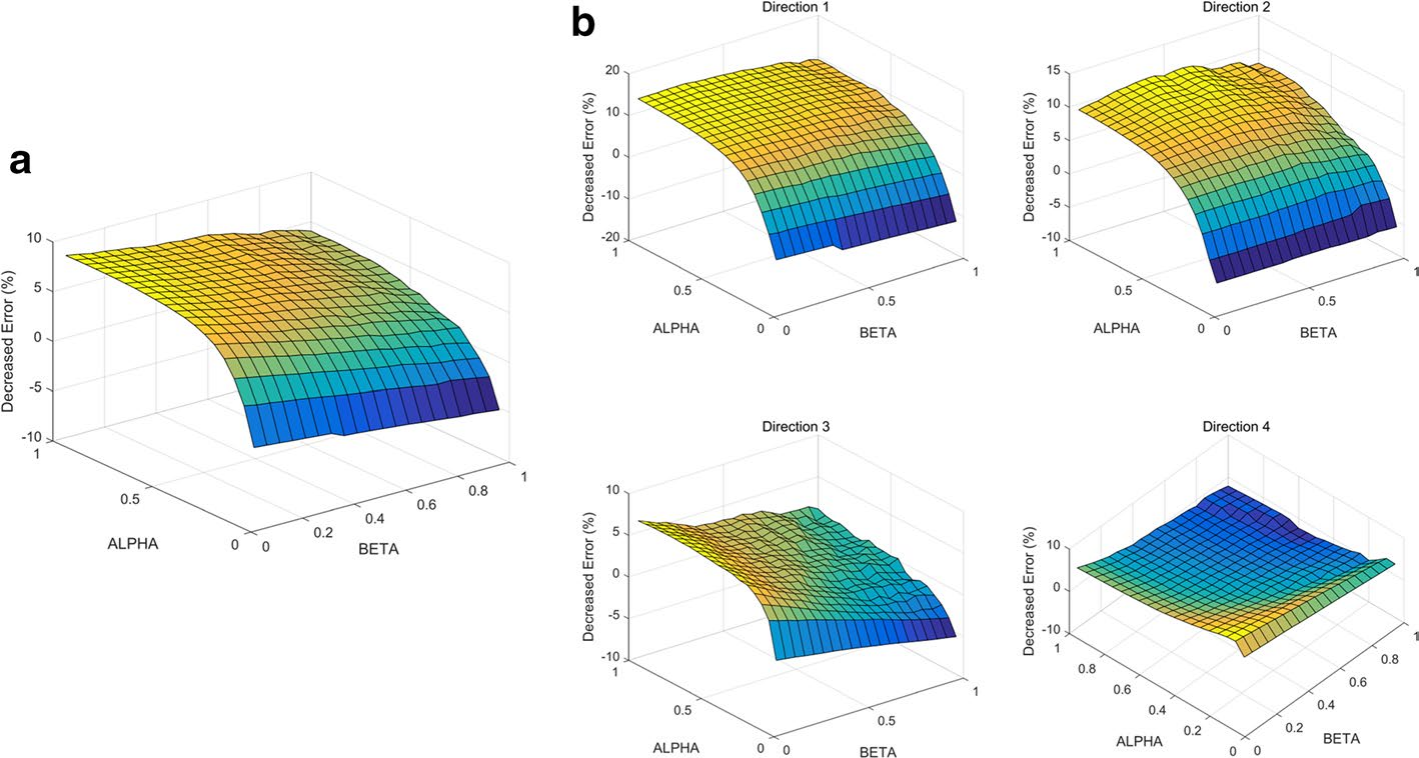
**a** Decreased error to the three nonintended targets averaged over 120 trials. **b** Decreased error to the three nonintended targets averaged over 30 trials for each direction. The surface graph has a larger value from blue to yellow

**Fig. 11:**
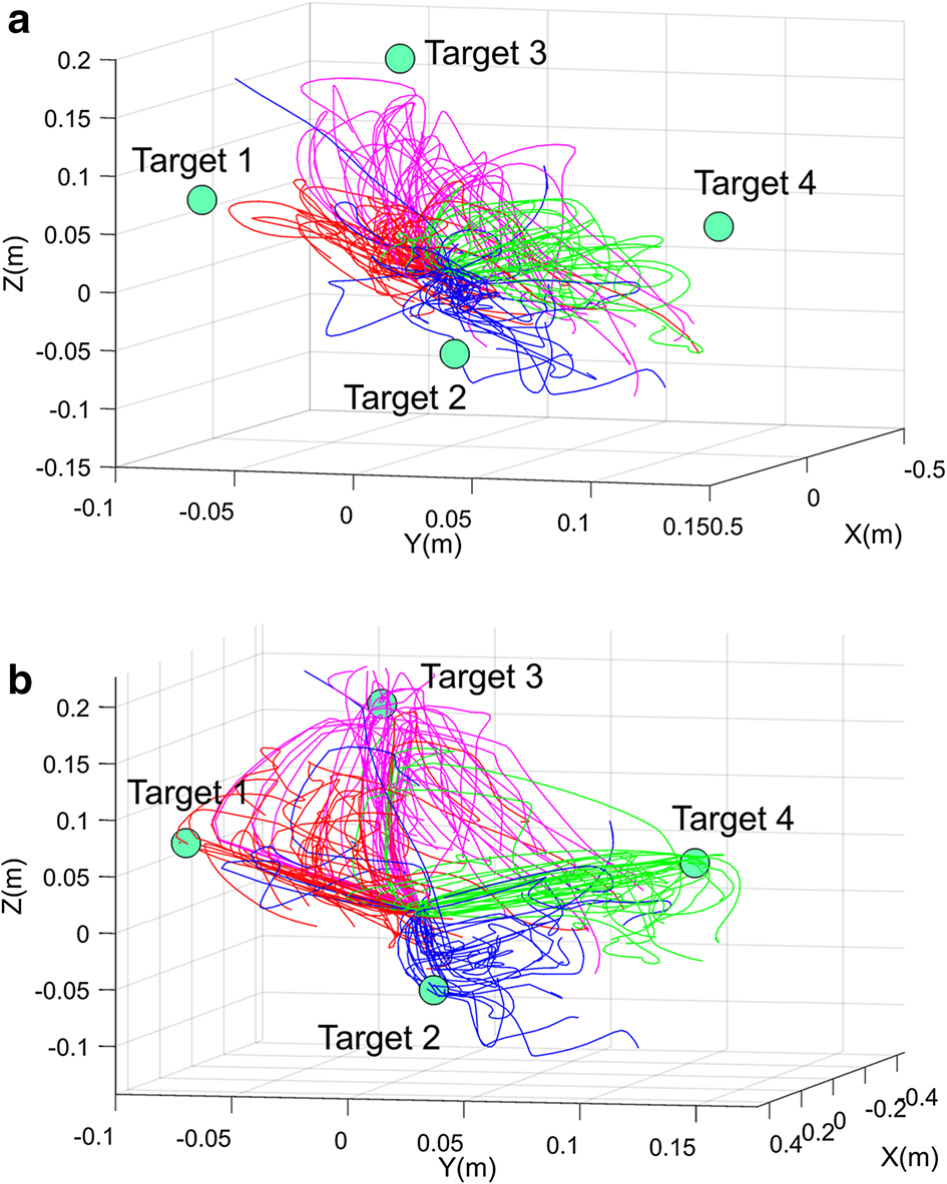
**a** Raw hand trajectories predicted from EEG. **b** Compensated hand trajectories using artificial potential. Red, blue, purple and green lines indicate predicted hand trajectory to reach targets 1, 2, 3, and 4 respectively

Whereas the implemented algorithm using artificial potential enabled the robot arm to more closely reach the intended target object, the shortest distance to nonintended target objects was not substantially affected by the algorithm. According to Fig. 10a, the average shortest distance to the nonintended targets decreased 4.07% when α = β = 1.00. The degree of improvement was affected by α rather than β. The condition α = β = 0.60 led to 5.84% improvement (Fig. 10b). In individual cases of each of the four directions, less than 15% improvement was observed. The decrease in the shortest distance to the nonintended target was significantly less than that of the shortest distance to the intended target. Thus, we confirmed that the algorithm enables the robot end-effector to selectively reach an intended target. The decrease in the shortest distances to the nonintended and intended targets were compared, as shown in Table 3. The *p*-value, calculated using a one-tailed two-sample t-test, implies that the distance error to the intended targets was significant less than to nonintended targets. Figure 11a, b show all the reaching trials before and after compensation, respectively (α = β = 0.60; 1 of the 120 total trials is not represented, as it is a serious outlier).

**Table 3:** Improvement of the predicted hand trajectory

α = β	Decrease in the shortest distance to intended target (unit: %, n = 120)	Decrease in the shortest distance to nonintended target (unit: %, n = 360)	*p*-value
1.00	57.37 ± 44.12	4.07 ± 29.18	***
0.90	55.62 ± 43.63	4.70 ± 28.67	***
0.80	54.73 ± 41.40	4.99 ± 26.22	***
0.70	53.97 ± 37.16	5.58 ± 23.47	***
0.60	51.85 ± 34.43	5.84 ± 20.40	***
0.50	49.15 ± 32.60	5.89 ± 17.51	***
0.40	46.63 ± 29.32	5.67 ± 14.74	***
0.30	41.98 ± 27.12	5.26 ± 12.48	***
0.20	33.02 ± 26.08	4.36 ± 12.27	***
0.10	8.34 ± 36.43	1.19 ± 19.02	*

*** *p *< 0.0001, * *p *< 0.01

### Effect of vision-aided BMI training in two clinical cases

**Fig. 12:**
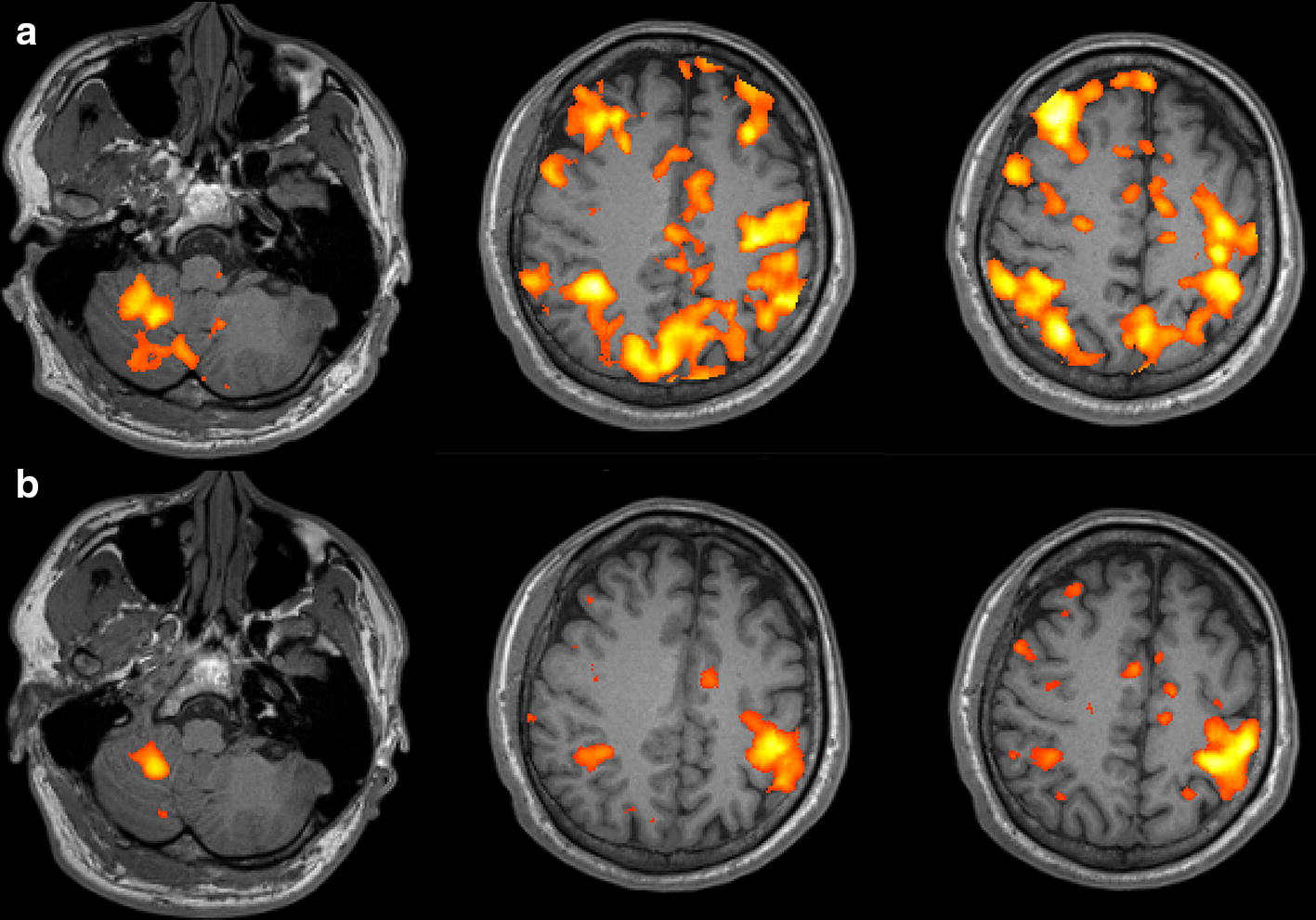
**a** fMRI image during reaching task (left arm) in participant #1 shows brain activation in various area including right primary motor and sensory cortex, posterior parietal cortex, prefrontal cortex, and left cerebellum (p < 0.001). **b** Brain activation is relatively focused to the right primary motor and sensory cortex, posterior parietal cortex, and left cerebellum after 5 sessions of BMI training with real-time visual feedback (p < 0.001)

Before the BMI training, both participants showed brain activation in multiple areas in both hemispheres in fMRI findings. Participant #1 demonstrated significant brain activation in the precentral gyrus (primary motor cortex), postcentral gyrus (primary sensory cortex), posterior parietal cortex (PPC), and lateral portion of the middle frontal gyrus and inferior frontal gyrus (prefrontal cortex) in the right hemisphere, which correspond to the left hand the participant was trying to move (Fig. 12a). The contralateral cerebellum was also significantly activated (Fig. 12a). After the training, brain activation was focused to the right precentral and postcentral gyri, PPC, and contralateral cerebellum (Fig. 12b). Participant #2 also demonstrated scattered brain activation across both hemispheres—especially in the occipital lobe—before real-time training; however, after training, the activated areas tended to focusing to the left precentral and postcentral gyri, PPC, and contralateral cerebellum, corresponding to the right hand the participant intended to move. However, in both cases, activation patterns did not differ between the three imagined reaching directions.

## Discussion

### Vision-aided BMI training system

The vision-aided system consists of an anthropomorphic robotic arm, Kinect, and aluminum profiles for fixing the other components. The Kinect enables target object detection and position estimation. In 60 validation trials (20 each for 2, 3, and 4 target objects), there were no failures in target detection. The implemented target detection algorithm can automatically detect multiple targets; however, it is still limited in that target objects must be green and the performance can deteriorate for increasing numbers of target objects. Improved image processing approaches, such as a convolutional neural network [33, 34], or a histogram of oriented gradients [35, 36], could potentially be applied as appropriate alternatives that overcome this issue.

The position estimation error rate was about 4.62%, and occurred for two main reasons. First, the calibration approach is linear. For camera calibration and position estimation, we applied linear regression using the least-squares method. Even though this approach performed satisfactorily, it cannot fully explain the complicated relations between variables. Second, the implemented algorithm estimated the center of two target objects, whereas the optic tracker measured the distance between stickers, causing differences between the two results. Furthermore, small errors caused by the internal Kinect software cannot be excluded.

### BMI system using artificial potential

Artificial potential, one of the main focuses of this study, was used to compensate for the joint movement of the robot arm and reach closer to the intended target. We found that the motion planning approach improved the shortest distance to the intended target up to 57.37%, whereas that to the nonintended targets showed almost no improvement. A t-test supported this observation statistically (*p *< 0.0001).

This result is meaningful because none of the information for determining the target object was preprogrammed. Although shared control based on high-level commands (a goal-oriented approach) frees the system from the burden of low-level and demanding high-speed interaction, it limits the robot arm to preprogrammed commands [37]. The suggested process-oriented approach provides low-level intervention in the robot arm movement and does not highly limit the paths by which the robot moves. The compensating information for following intended target was obtained from only raw hand velocities predicted from EEG and the positions of possible targets. Thus, the suggested algorithm maintains the volition of the user and improves robustness and accuracy.

### Optimal blending parameters

When α = β = 0.60, the degree of improvement is sufficiently saturated, such that the shortest distance to the intended target decreased by 51.85%, which is approximately 90% of 57.37%. According to Table 4, the degree of improvement sufficiently saturated at the condition α = β = 0.60. In the case of Direction 4, the condition in which α = β = 0.60 (60.49%) exhibited greater improvement than the condition in which α = β = 1.00 (55.28%); therefore, we infer that blending parameters exceeding 0.60 are not highly effective.

**Table 4:** Improvement of the predicted hand trajectory at α = β = 0.60 and 1.00

(α, β)	Direction 1 (%)	Direction 2 (%)	Direction 3 (%)	Direction 4 (%)	Average (%)
(1.00, 1.00)	41.12	51.20	81.90	55.28	57.37
(0.60, 0.60)	36.20	37.18	73.55	60.49	51.85

Less blending parameter α provides less compensation and higher decoding power of user and decoder should be guaranteed. The blending parameter α can be modulated to sustain the success rate of instructed tasks and motivate the users [11]. Low blending parameter β implies that the robotic arm reflects the previous velocity vector value as high weight, and sudden unpredictable motions are sufficiently suppressed. The parameter β can be determined by considering the user safety and signal characteristic. When it is considered that user prefers BMI system with high level of user volition and safety, α = β = 0.60 can be provided as the optimal condition if initial decoder.

### Advantages of novel BMI training system

The suggested training system preserves the high level of user volition for the robotic arm control. It gives the training system the following advantages. First, it is easy to set the appropriate chance level for active shared control. Since active shared control can reach a target even with random walk signal through an auxiliary control command, reaching the target point is not a measure of success. Rather, it is a more objective indicator of success to reach the intended target among various candidate objects. In this case, the chance level is calculated as in Eq. (22).

22 $$ Chance\;\;Level  \, \left( {\text{\%}} \right) = 100 \div Number  \, of  \, Targets $$

Second, it is easy to perform robust parameter training because the targets can be moved by using flexible cable as used in the current study. Trained model parameters for targets at various locations can avoid the issue of the decoding model being over-fitted to objects at specific locations. In addition, the reaching success rate of the target object at any arbitrary position can give a higher sense of trust to the reader of a paper or the viewer of a video.

### Effect of vision-aided BMI training in clinical application

In the first phase of training (first 5 training sessions), the participants were given a VR video of robotic motion for motor imagery of reaching motion, as the purpose of this phase was to accustom them to the imagery task. Users were instructed to assume that the robotic arm was their arm, as the robotic arm was positioned nearest to their paralyzed arm. However, even after 5 sessions of imagery training, fMRI revealed that brain activation areas were scattered across motor planning and execution areas in both hemispheres. This result suggested a less characterized pattern of brain activity, although the most significantly active areas were consistent with previous studies reporting areas activated during motor imagery and BMI learning: the ipsilateral premotor cortex, supplementary motor area, primary motor cortex, PPC, insula, and contralateral cerebellum [38, 39].

After 5 additional training sessions with the observation-based training provided, brain activation patterns tended to focused to the primary motor and sensory areas, PPC, and contralateral cerebellum. Wander et al. [40] also demonstrated that cortical adaptation occurs during BMI learning, during which activation in the prefrontal cortex, premotor cortex, and PPC tend to decrease as users switch from cognitive to automatic phases. However, in our study, whereas prefrontal and premotor cortex activity decreased significantly, PPC activity was relatively sustained. This difference may arise from the fact that the participants were still in the training phase of external robot control, which may require PPC and cerebellar activity for planning movements and BMI learning [41], even if imagining the reaching movement itself may have entered the autonomic phase, requiring less prefrontal and premotor activity. This result suggests that reorganization and plasticity of the brain play an essential role in BMI training, in addition to algorithm-based training of the system, such as machine learning methods. Cortical reorganization during BMI training for reaching and grasping or neurofeedback in rehabilitation has also been shown in other studies [42, 43]. Because the gross activation pattern significantly changes with repeated training, machine learning parameters should be updated with each training session.

### Limitations

Using the proposed shared control strategy for the robot end-effector to reach the intended target, the success rate of reaching the instructed target did not exceed the chance level significantly. It is possible that, as we could not confirm the difference in brain activation according to the direction of motion imagery in fMRI, it may be necessary to observe the activation with electrodes of a higher spatial resolution in the area where activation is focused. At least, it is almost certain that EEG cannot distinguish the pattern inside the active area of the fMRI image in Fig. 12b. So, as the future study, more invasive electrodes with finer spatial resolution can be considered. Electrodes for electrocorticography, which is less invasive (intermediate type of invasive and non-invasive electrodes) is expected to provide improved results.

## Conclusions

This study presents a BMI training system that automatically detects and estimates the position of possible target objects. Further, we applied an artificial-potential-based algorithm to predicted hand velocity, and it enabled a robot arm to selectively reach for an intended target. The developed training system was applied to two potential users with cervical spinal cord injury, and provided observation-based training. The pilot clinical study utilizing the training system suggested potential beneficial effects in characterizing the brain activation patterns. The suggested training method may help adaptation and optimization of the brain activity, to eventually utilize the BMI practically as an assistive technology. However, the shared control was not successful because of low decoding power. When the size of focused activation area is considered, finer electrode with higher spatial resolution is required. The suggested training method may help adaptation and optimization of the brain activity.

## Abbreviations

BMI: brain–machine interface
DOF: degree of freedom
MEA: microelectrode array
EEG: electroencephalography
fMRI: functional magnetic resonance image
AIS: American Spinal Injury Association Impairment Scale
PPC: posterior parietal cortex
ADL: activities of daily living
